# Occurrence and Fate Analysis of Mycotoxins in Maize During the Post-Harvest Period

**DOI:** 10.3390/toxins16110459

**Published:** 2024-10-26

**Authors:** Yajie Zheng, Wenfu Wu, Changpo Sun, Hujun Liu, Jianpeng Dou

**Affiliations:** 1 College of Biological and Agricultural Engineering, Jilin University, Changchun 130022, China; zhengyjss@126.com (Y.Z.); wwfzlb@126.com (W.W.); lhj@ags.ac.cn (H.L.); 2 Academy of National Food and Strategic Reserves Administration, Beijing 100037, China; chpsun@163.com

**Keywords:** maize, post-harvest, mycotoxins, occurrence, fate analysis

## Abstract

The consumption of agricultural products contaminated with mycotoxins poses a significant threat to the health of both humans and animals. Maize frequently becomes contaminated with toxic fungi while it is still growing in the field. Therefore, more proactive measures should be implemented to reduce mycotoxin levels during the storage and processing of maize after harvest. This article analyzes the prevalent mycotoxins found in maize, specifically aflatoxins, ochratoxins, trichothecenes, fumonisins, and zearalenone. The study provides a comprehensive analysis of the occurrence of mycotoxins in maize during storage, as well as fate analysis of them during processing. It summarizes the impacts of storage time, environmental conditions, storage methods, and agricultural practices on mycotoxin occurrence during storage in the post-harvest period. Furthermore, the different distribution of mycotoxins across various fractions during both dry- and wet-milling processes in the post-harvest processing period is analyzed. Additionally, the strategies to control mycotoxins in maize are also proposed during the post-harvest period. This review offers valuable insights for future research on mycotoxin contamination in maize during the post-harvest period.

## 1. Introduction

**Figure 1:**
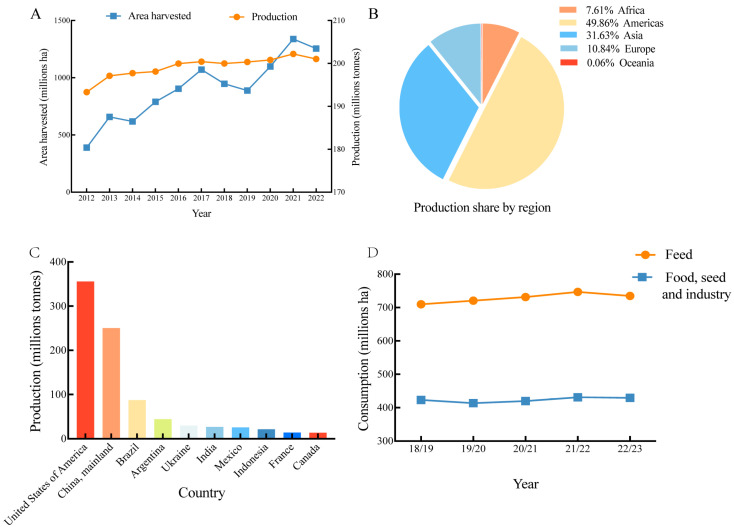
Global maize production from 2012 to 2022. (**A**) Production levels (million tonnes). (**B**) Production share by region. (**C**) Top 10 global maize-producing countries (million tonnes). (**D**) Global maize consumption from 2018/2019 to 2022/2023 (million tonnes). Data from FAO [2] and USDA [1].

Maize, a monoecious cereal belonging to the Poaceae family, is a crop of global importance. It is abundant in starch, proteins, fats, vitamins, and various other essential nutrients. Maize undergoes deep processing, such as starch production, which yields components essential for living organisms. Maize is widely cultivated worldwide, attaining the highest total yield among all crops, particularly in the United States and China. According to a report, U.S. maize production is projected to reach 14,888 billion bushels in 2023, representing an increase of 1158 billion bushels from 1373 billion bushels in 2022. For the year 2024, maize production in the United States is anticipated to reach approximately 1499 billion bushels, with estimates varying between 1458 billion bushels and 1540 billion bushels. These projections are derived from the USDA’s end-of-June acreage report and the crop conditions as of June 30. Since 1985, the maize-production industry in China has undergone significant growth. Data from the National Bureau of Statistics indicate that China’s maize production reached 288,842 million tonnes (approximately 57,768 billion pounds) in 2023, marking an increase of 2328 billion pounds year-on-year, with a growth rate of 4.2%. Forecasts by the Ministry of Agriculture and Rural Development, in collaboration with associated agencies, project that maize production for the 2024/25 season will reach 29,701 million tonnes. Overall, global maize consumption remains stable, with feed consumption, as a crucial source of nourishment for the international livestock industry, exhibiting a generally upward trend on an annual basis. Furthermore, as the global livestock industry expands, the demand for maize as feed is anticipated to rise continually. According to data reported by the United States Department of Agriculture (USDA), the global consumption of maize for feed purposes amounted to 762.23 million hectares in the 2022/2023 marketing year, and consumption for food, seed, and industrial uses totaled 443.83 million hectares [1]. The global production levels of maize, the production share by region, the top ten maize-producing countries worldwide, and the global maize consumption from 2018/2019 to 2022/2023 are illustrated in Figure 1. Maize is cultivated extensively worldwide, and a substantial proportion of it is contaminated with mycotoxins.

Mycotoxins are secondary metabolites synthesized by a range of saprophytic fungi, notably including the genera *Aspergillus*, *Penicillium*, and *Fusarium* [3]. These compounds are classified as low molecular weight molecules [4,5] . The principal mycotoxins encompass aflatoxins (AFs), ochratoxins (OTs), trichothecenes (TRCs), fumonisins (FBs), and zearalenone (ZEN), along with their respective metabolites. A study on maize production in South Africa revealed that 83% of the collected samples contained mycotoxins, with 50% of these samples exhibiting multiple mycotoxins, including deoxynivalenol (DON), 15-acetyl-deoxynivalenol, diplodiatoxin, and ZEN [6]. Maize samples designated for human and animal consumption in Vietnam exhibited contamination with elevated concentrations of aflatoxin B_1_ (AFB_1_) and fumonisin B_1_ (FB_1_) [7]. Recent analyses of maize samples collected for multiple mycotoxin assessments between 2018 and 2020 revealed that 18% of the 480 samples obtained from Kenyan households exhibited ZEN levels exceeding 1000 μg/kg [8]. Furthermore, the dissemination of DON in maize harvested in Ohio and Indiana during the autumn of 2022 significantly affected the maize yield in those states [1]. It is evident that mycotoxin contamination in maize not only impacts the national economy but also presents substantial health risks to both humans and animals that consume maize-based products [5]. Mycotoxins exhibit significant thermal and chemical stability, which renders them difficult to eliminate [7,9].

Mycotoxins may be produced and accumulate during the harvesting, storage, and processing of maize following its maturation in the field [10]. The occurrence of mycotoxins can be influenced by several factors, including the timing of storage, the methods of storage, the environmental conditions, and the agricultural practices during storage. Processing, an essential phase in various food supply chains, can substantially affect the levels of mycotoxins. The assessment of mycotoxin behavior during both dry- and wet-milling processes in maize is crucial for managing contamination and improving processing methodologies [11]. Therefore, it is imperative to investigate the occurrence and fate of mycotoxins in maize during the post-harvest period.

This review provides a comprehensive summary of the occurrence and fate of mycotoxins in maize during the post-harvest period. This study presents the common types of mycotoxins identified in maize and analyzes their occurrence during storage. Additionally, the fate of mycotoxins during both the dry- and wet-milling processes is analyzed. This review aims to guide future studies on mycotoxins in maize.

## 2. Mycotoxins in Maize

AFs are toxic secondary metabolites primarily produced by the fungus *Aspergillus flavus* [12], which consists mainly of AFB_1_, AB_2_, AFG_1_, and AFG_2_. AFs have been widely reported globally, with the highest incidence occurring in Africa, followed by Asia, Europe, and the Americas [13]. In the field, maize kernels are susceptible to colonization by the conidia of *Aspergillus flavus*. The interactions between soil organisms and rainwater contribute to the establishment of conditions that are conducive to the growth and proliferation of *Aspergillus flavus* in maize [14]. Studies have revealed that *Aspergillus flavus* is associated with the hot and rainy climate of African countries, which favors the growth of *Aspergillus flavus* as well as the production of AFs [13]. Unfavorable environmental conditions during the harvesting and storage processes can contribute to the proliferation of AFs. The contamination of maize by AFs leads to significant economic losses and poses serious risks to food and feed safety, as well as to consumer health. Numerous studies have reported that AFs exhibit teratogenic, immunosuppressive, and mutagenic properties [15], with AFB_1_ identified as the most common and potent carcinogen, it has been classified as a Group 1 carcinogen by the International Agency for Research on Cancer (IARC) [16,17].

OTA is produced by both *Aspergillus* spp. as well as *Penicillium* spp. and is widely accepted, including OTA, OTB, and other mycotoxins [18]. OTA, recognized as the most toxic class of fungal metabolites to animals, is commonly detected in food crops, particularly maize, during the flowering phase and the initial stages of kernel development [19]. In a study of maize samples from Pakistan, OTA was detected in 61% of the maize kernel samples across various varieties, with 22% of these samples surpassing the permissible limit. The mean concentrations of OTA in maize kernel samples varied between 1.41 and 53.9 μg/kg [20]. The consumption of food by humans and animals presents considerable health risks, particularly about renal toxicity and the immunosuppressive properties associated with OTA [21,22].

Fusarium toxins, including TRCs such as DON, nivalenol (NIV), T-2 toxin (T-2), HT-2 toxin (HT-2), as well as FBs (FB_1_ and FB_2_), and ZEN, are frequently detected in maize, often at high levels of occurrence and concentration [23]. Research has indicated a possible correlation between TRCs and ear rot in maize [24,25]. *Fusarium verticillioides* is the primary fungal pathogen implicated in the etiology of ear rot [26]. ZEN, also known as F-2 toxin, is a mycotoxin produced by *Fusarium roseum*, the anamorphic stage of *Gibberella zeae*, and is associated with maize ear rot [27,28,29]. Maize exhibits a high susceptibility to contamination by ZEN [30,31,32]. A study conducted in Asia in 2019 revealed that 75% of maize samples were contaminated with ZEN. The consumption of maize-based foods with high levels of ZEN can lead to hyperestrogenism and diseases related to cervical cancer in humans [33,34]. Global surveys conducted in 2017 revealed that approximately 75% of raw maize and 35% of maize-based food products were found to contain FBs [35]. In June 2024, Anyou Company, located in China, conducted tests on a total of 2782 feed samples. The findings revealed that 5.5% of the maize samples exceeded the DON threshold, with the maximum concentration detected being 7900 µg/kg.

In addition to the commonly referenced mycotoxins, maize may also be susceptible to contamination by a variety of other mycotoxins. One notable mycotoxin is alternariol, which is produced by *Alternaria*, and widely contaminates cereals (including maize) and feed. The secondary metabolites of alternariol may exert both chronic and acute toxic effects on humans and livestock, which include mutagenicity, carcinogenicity, and teratogenicity [36,37]. Citrinin (CIT), a mycotoxin produced by various species of *Penicillins* and *Aspergillus*, has also been identified in maize. The substance has the potential to induce hepatotoxicity and nephrotoxicity across various animal species [36]. Additionally, it has been demonstrated to inhibit RNA, DNA, and protein synthesis in pigs [38]. The mycotoxins are also regarded as the primary emerging mycotoxins in maize [39,40]. Table S1 delineates the various types of common mycotoxins found in maize.

## 3. Occurrence of Mycotoxins in Maize During the Post-Harvest Period

### 3.1. Pretreatment

#### 3.1.1. Transporting

The transporting of maize prior to storage has a significant impact on mycotoxin contamination [41]. Zhang et al. observed that the contents of DON and F-2 decreased significantly during short transportation times (less than 200 min) [42]. Another study found that AFs can accidentally be produced during maritime transport under conditions of high temperatures and humidity, specifically, AFB_1_ production reached 100 µg/kg in 5% of the batch during ship transport [43]. Furthermore, inadequate transportation practices may result in damage to maize kernels, which can subsequently facilitate the development of mycotoxins.

#### 3.1.2. Drying

The moisture content of maize kernels exhibits variability, and maize requires timely drying after harvest. The study indicated that moisture levels during the initial stages of maize harvest were approximately 13% in both 2013 and 2015, the drying conditions at these levels were not favorable for the synthesis of specific *Fusarium* species. This is evidenced by the frequency of DON contamination in maize, which was 2.5% and 15.5%, respectively [44]. Research has demonstrated that maize kernels exhibiting a moisture content ranging from 28% to 33% at harvest are conducive to DON and NIV growth and the subsequent production of various mycotoxins [45].

#### 3.1.3. Cleaning

Cleaning involves the removal of foreign material (such as stones, weed seeds, and maize cob fragments) as well as low-quality maize kernels (small, broken, or crumpled ones) [11]. Before storage, cleaning maize helps prevent fungal infections and limits the further spread of mycotoxins. Numerous studies have demonstrated that manual sorting and washing/floating of maize kernels can lead to mycotoxin reductions of 50% or more [46,47,48]. For instance, when Kenyan households visually sorted and hand-washed their maize in water for 10 min, FBs contamination was reduced by an average of 65% [48]. In a study by Scudamore and Patel, one maize batch showed little to no reduction in total FBs concentration after initial cleaning, while the other 10 batches exhibited a reduction of over 16%, with an average reduction of approximately 34% (overall average of about 30%) [49]. Increasingly sophisticated cleaning technologies are being developed [50,51]. One study introduced a rotating brush to experimentally clean maize by brushing off dust and broken kernels through a screen. This technique resulted in reductions ranging from 57% to 99% for AFB_1_ and from 36% to 100% for AFB_2_ [52].

### 3.2. Storage

#### 3.2.1. Storage Time

The duration of maize storage is correlated with the production of AFs [53,54,55]. A study examining AF contamination in maize stored by traders in Uganda found that maize stored for more than six months had significantly higher AF levels than maize stored for 2 to 6 months. Specifically, the mean AF content was 30.2 ppb at mid-altitude (moist), 22.5 ppb at mid-altitude (dry), and 12.8 ppb at highland for maize stored for more than six months. In contrast, the mean AF content was 20.54 ppb, 18.02 ppb, and 12.35 ppb, respectively, for maize stored for 2 to 6 months [56]. Hell et al. reported that at the start of storage, 2.2% to 5.8% of samples had AF levels exceeding 100 ppb. After six months of storage, the percentage of samples with these high levels increased to a range of 7.5% to 24% [53]. Similarly, Hell et al. observed an increase in the percentage of samples with high AF levels from the beginning of storage to six months later. These AF-positive samples had initial levels ranging from 22 to 190 ppb, which increased to 31 to 221 ppb after six months [54].

#### 3.2.2. Storage Methods

The method of storage employed for maize has a significant impact on the prevalence of mycotoxins. The study revealed that maize stored in polypropylene bags demonstrated significantly lower levels of FB_2_ contamination (mean FB_2_ = 38 mg/kg) compared to maize stored without bags (mean FB_2_ = 99 mg/kg) [57]. Furthermore, a reduced number of fungal species capable of producing mycotoxins were identified in maize kernels stored in triple-layer polyethylene hermetic bags in comparison to those stored in standard polypropylene (PP) bags. Polyethylene fibers exhibit enhanced resistance to bending and abrasion when compared to polypropylene fibers. The levels of AFs in maize stored in Purdue Improved Crop Storage (PICS) bags and polypropylene (PP) bags were observed to differ. Specifically, at 12, 16, and 24 weeks of storage, the AF levels in PP bags were 2.43 ppb, 2.71 ppb, and 0.6 ppb, respectively. In contrast, the AF levels in PICS bags were 3.15 ppb, 1.82 ppb, and 0.33 ppb, respectively. These results indicate that PICS bags had a better ability to inhibit the production of AFs at longer storage times (16 and 24 weeks) compared to PP bags [58]. Furthermore, the increase in AF content in maize stored in conventional PP bags was significantly greater than that observed in various sealed storage devices, including plastic and metal containers. The study in Meru found that maize stored in conventional PP bags had a significant increase in total AF levels when subjected to dry treatment, with a 117% increase compared to only 20% and 25% increases in AF levels for maize stored in plastic silos and metal silos, respectively. Similarly, wet-treated maize stored in conventional PP bags showed a 116% increase in total AF levels, while the increase was only 15% and 16% for maize stored in plastic silos and metal silos, respectively [59]. Additionally, the study found that the levels of mycotoxins were significantly reduced when grains were stored in silo bags compared to trench silos. Specifically, AFB_1_ was detected in samples from the trench silo ranging from 1 to 160 µg/kg, while silo bag samples were contaminated with AFB_1_ levels ranging from 5.8 to 47.4 µg/kg. Furthermore, FB_1_, ZEN, and PAT were not detected in any of the silo bag samples [60]. The susceptibility of maize to environmental conditions is attributed to the placement of trench silos on exposed soil [61]. Silo bags are more effective for the storage of maize as they facilitate the maintenance of a low concentration of O_2_ and a high concentration of CO_2_ within the hermetic environment of the bag [62,63,64]. A study examining maize stored in a ventilated crib, a metal bin, and bags in warehouses revealed that the bagged maize contained significantly higher levels of AFs compared to the other storage methods. The levels of aflatoxins (AFs) in bags stored in a warehouse were found to be 6.5 ppb in October, 22.2 ppb in December, and 9.0 ppb in April. In contrast, the levels of AFs in cribs and metal bins were lower, ranging from 2.0 to 3.4 ppb in October, 8.4 to 14.7 ppb in December, and 4.1 to 6.0 ppb in April [65]. Additionally, various maize-storage methods have been examined, including storage under the roof, in “Secco” (a giant basket made from Hypparhenia diplandra), the “Zingo” (a granary with a wooden conical base), and in the “Ago” (a giant basket made from woven raffia palms). Higher levels of AF contamination were identified through the application of all these methodologies. The inadequate aeration in “Ago” facilitates the proliferation of fungi, leading to considerable mycotoxin contamination [53]. The risk of AF contamination is elevated when maize is stored in clay storage facilities, as humidity can accumulate through convection, enabling *Aspergillus* spores to persist for prolonged durations under such conditions [53].

Recent research has indicated that the hermeticity of storage facilities is essential for mitigating the risk of mycotoxin contamination in maize. The levels of mycotoxins in maize kernels exhibited relative stability throughout a six-month storage period within hermetic systems. In contrast, maize stored in conventional non-sealed systems demonstrated a consistent increase in mycotoxin levels over time [59,66]. Additionally, it is imperative to engage in ongoing evaluation and enhancement of sealed storage bags to improve the management of mycotoxins during the storage of maize.

#### 3.2.3. Storage Environmental Conditions

Environmental conditions, including relative humidity, temperature, and carbon dioxide concentrations, play a critical role in the formation of mycotoxins during storage. The optimal temperature range for the production of AFB_1_ during storage is identified as being between 25 °C and 35 °C [67]. Elevated temperatures promote the proliferation of *Aspergillus flavus* and enhance the production of AFs. The threshold of water activity (aw) for the development of various mycotoxins is a critical factor in assessing the risk of mycotoxin contamination. For instance, aw significantly impacts on AFB_1_ contamination, as *Aspergillus flavus* does not exhibit prolonged growth at an aw of 0.85 [68], thereby preventing high levels of AFs in maize [69]. For OTA, it is essential to maintain storage conditions that ensure a relative humidity of 50% and an ambient temperature of 20 °C to keep OTA levels generally low [70]. Furthermore, increased levels of relative humidity, temperature, and carbon dioxide (CO_2_) in storage barrels led to a 20–40% increase in the content of FBs during the storage period [71]. Research has shown that the susceptibility of maize kernels to the proliferation of *Fusarium verticillioides* increases when the CO_2_ concentration is elevated to 800 ppm. However, elevated CO_2_ concentrations decrease FBs per unit of functional biodiversity. The optimal storage conditions for the formation of FBs include a temperature of approximately 30 °C, an aw of 0.98, and a concentration of 400 ppm of CO_2_ [72]. The highest concentrations were observed at an average temperature range of 18.8 to 21.48 °C, with moisture content levels between 12.6% and 16.8%, and relative humidity values ranging from 80.6% to 83.2%. The study found that Fusarium toxins exhibited a significant negative correlation with average temperature and relative humidity, while demonstrating a significant positive correlation with seed moisture content [73].

In contrast to field infestations, the majority of insect infestations and colonization take place during the post-harvest process [74]. Mycotoxin contamination in stored maize, which is often attributed to pest infestations [75]. The contamination of agricultural products by aflatoxins during storage is closely linked to elevated weevil density [76], and is generally linked to suboptimal storage conditions. Grain silos can serve as habitats for pests that reproduce more rapidly in elevated temperatures, and contribute to the establishment of a microclimate that facilitates fungal proliferation, leading to increased production of metabolic water. The increase in condensation, along with the presence of wet spots, may enhance the activity of spoilage fungi. This phenomenon has the potential to elevate contamination levels of mycotoxins, including OTA, AFs, and possibly TRCs in damp grain [77]. The study demonstrated that the infection rate of AFs in maize infested with insects was 87%, in contrast to 25% in samples that were free of insects [53]. And the contamination of AFs is also directly correlated with the population size of insects. Moreover, insect infestations significantly enhance the production of mycotoxins when CO_2_ concentrations exceed 400 ppm and relative humidity levels surpass 80%. An analysis of the underlying causes suggests that insects can cause damage to maize cobs, thereby compromising the natural defenses that protect the maize and increasing the area susceptible to fungal colonization [78].

#### 3.2.4. Storage Agricultural Practices

It is imperative to clean and sanitize the storage equipment when preparing maize grains for storage. Additionally, the equipment must be equipped with an effective aeration system and temperature sensors to ensure optimal storage conditions [77]. A proper covering of maize cobs significantly reduces AF contamination, as an effective husk serves to prevent insect damage and water infiltration [53]. The frequent turning of maize during storage has been associated with an increase in DON contamination. This phenomenon is likely due to the ventilation that occurs during the turning process. It is now widely acknowledged that stored maize should undergo regular ventilation, especially in hot and humid conditions. A study has indicated that maize possesses a moisture content of 20%, necessitating adequate aeration for prolonged storage. If the material is not subjected to aeration, this factor may lead to contamination by OTA [79]. However, the introduction of ventilation may result in the influx of cold air, thereby reducing the temperature within the microclimate surrounding maize. This temperature reduction in turn increases the risk of mycotoxin contamination [41]. Additionally, the co-storage of crops may affect the development of mycotoxins. Maize stored in the same warehouse as sorghum has been associated with elevated levels of AFs [53]. High levels of mycotoxin contamination in maize may arise if the co-stored crop is also contaminated with mycotoxins. A study identified elevated levels of aflatoxin contamination in maize stored alongside cowpeas, which are known to be susceptible to aflatoxins in agricultural settings [80].

## 4. Fate Analysis of Mycotoxins in Maize Processing During the Pre-Harvest Period

Maize starch, maize sugar, maize oil, and other valuable products can be obtained through various deep processing techniques, with the wet-milling process demonstrating notable effectiveness. This process enables the extraction of the nutrient-rich germ, as well as the pericarp and endosperm, which are critical components for further refining into a variety of products. These components are predominantly employed in the production of animal feed and for human consumption. Dry-milling is a fundamental processing step that establishes a foundational raw material for subsequent processing stages. Maize flour obtained through the dry-milling can be utilized as a raw material for producing deep processed products, including maize noodles and maize biscuits. Therefore, investigating the behavior of mycotoxins throughout the milling process yields critical insights for their effective management [81]. Tables S2 and S3 summarized the fate of mycotoxins in maize dry-milling and wet-milling process.

### 4.1. Dry-Milling

Dry-milling involves the physical removal of the surface portion, such as the hull and bran, while minimizing endosperm breakage to produce desired grits, meals, and flours. These products are primarily used as raw materials in feed and food production [82]. Although mycotoxins in contaminated maize are not destroyed, their levels may be redistributed among the various dry-milling fractions and products, potentially resulting in higher or lower concentrations. Therefore, it is crucial to evaluate the impact of dry-milling on mycotoxin redistribution. Furthermore, research indicates that the redistribution of mycotoxins in the maize dry-milling fractions depends on the variety, milling method, and the specific fractions produced in different countries [82]. Investigating the fate of mycotoxins during dry-milling will help mitigate the economic impact of mycotoxins in the maize production chain and assess the actual risks to human and animal health.

#### 4.1.1. Aflatoxins

Researchers observed a reduction in AFB_1_ content in the endosperm fraction, although this reduction was not statistically significant. Conversely, AFB_1_ levels increased significantly in the bran and germ fractions, with increases ranging from approximately 2 to 9 times. The AFB_1_ levels in maize meals and grits were found to be low [83], as these products are derived from the endosperm, the inner part of the maize is less contaminated with AFs. Another study reported that the retention of AFB_1_ in grits was 13% of the initial concentration, while the bran and germ fractions exhibited increases of 379% and 629%, respectively, following dry-milling of maize with an initial AFB_1_ content of 91.1 µg/kg [84]. A study showed that AFB_2_ was present in 74% of the endosperm, 16% of the pericarp, and 9.9% of the germ [85]. Overall, there are relatively few studies on AFs in industrial dry-milling of maize, some of which include a cleaning step. Additionally, there was significant variation between batches [84,86]. Varying detection techniques also contribute to differences in the mycotoxins in the various fractions.

#### 4.1.2. Fusarium Toxins

High levels of FBs were detected in animal feed flour produced through dry-milling [83]. There was a 167% increase in FB_1_ contamination in bran, while coarse and fine grits exhibited 90% and 73% lower FB_1_ levels, respectively, compared to clean maize with an initial FB_1_ content of 8841 µg/kg [84]. Mold growth typically occurs from the surface to the interior of the maize, leading to the accumulation of FBs primarily in the surface layers [87]. Young et al. found that mycotoxins are generated at the site of fungal growth and are not transported from the surface to the interior of the kernel [88]. The germ, located on the outside of the maize kernel, is rich in fat, which facilitates mold infestation and mycotoxin production [89]. The low levels of FB_1_ in maize meal and flaking grits can be attributed to the bran layer acting as a physical barrier, preventing further penetration of mycelia into the inner kernel structure [83,90]. However, minimal breakage of the endosperm also results in reduced transfer to the inner kernel. The concentrations of FBs in flour fractions were significantly and positively correlated with those in unprocessed maize.

In addition, the contamination of FBs in the dry-milled maize fractions was correlated with particle size. Bennett et al. found that the content of FBs in pearl meal and break meal was notably lower than in maize flour. Compared to flaking grits, medium and small hominy grits were 1.9 and 3.6 times more contaminated, respectively [91]. Bordini et al. reported higher levels of FB_1_ in maize meal (mean values of 168.24 and 177.52 μg/kg for the two batches, respectively) than in grits (89.15 and 41.99 μg/kg, respectively). The endosperm likely consists of both horny and softer fractions, with the softer fractions containing higher concentrations of FB_1_ and being heavily contaminated. Maize flour is typically extracted from these softer fractions [82]. Grits, on the other hand, are usually derived from the hard endosperm and are considered less contaminated due to their hardness. Pereira et al. also found higher levels of FB_1_ in fine grits and fine flours compared to the coarse fractions of the dry-milled maize [5,86,90]. Additionally, differences in the distribution of FBs in maize fractions arise from various industrial dry-milling processes [91,92].

The fate of ZEN during the dry-milling of maize shows higher concentrations in the hull and high-fat fractions, while lower concentrations are observed in grits, low-fat meals, and flour [91]. Romer reported that with an initial ZEN concentration of 1000 µg/kg in raw maize, the ZEN concentrations in the endosperm-based fractions (grits, meal, and flour) were 50, 75, and 950 µg/kg, respectively. In contrast, the ZEN concentration in the screenings and germ were significantly higher, at 8990 and 4000 µg/kg, respectively [93]. However, another study found higher levels of ZEN in the flour compared to the raw maize batch [94]. This discrepancy may be attributed to the sampling procedure in the industrial plant process, where the sub-components may differ from the raw materials entering the process [95]. For DON, the germ is about 4 times higher than the initial maize, while the grits are 3 times lower [94].

The concentration of trichothecenes is reduced by approximately 50–90% in products for human consumption obtained through the traditional dehulling process [96]. Additionally, studies on other mycotoxins (NIV, FUS-X, T-2, Neosoloaniol, T-2 triol, T-2 tetraol, HT-2, 4,15-diacetoxyscirpenol, 15-monoacetoxyscirpenol, scirpentriol) found that except for NIV, FUS-X, and T-2 toxins, other trichothecenes were not detected in the grits and flour. Several mycotoxins were discovered in the bran, germ, germ meal, or screening material [94]. Furthermore, increased concentrations of 3-acetyl-DON, 15-acetyl-DON, and FUS-X were observed in industrial dry-milling by-products, where they were found in some batches of raw maize at low levels, often associated with DON levels [97].

Fusarium toxins exhibit similar behavior to AFs during the dry-milling of maize, with both showing lower levels in the endosperm and higher levels in the bran and germ fractions [83,86,90]. The available data also reveal greater variation in mycotoxin levels in the germ fraction. Specific studies may take different approaches and exhibit variations due to different milling techniques [86,87,90]. Therefore, a summary of similar distribution trends of fusarium toxins in different dry-milling fractions was conducted.

Different types of mycotoxins generally contaminate various parts of maize to varying degrees during dry-milling. The differences in mycotoxin content among the fractions obtained from dry-milling are related to the yield of each fraction [82]. Bran and germ, which have lower industrial yields, exhibit the highest levels of FB contamination. This can be explained by the relatively low amount of contaminated maize compared to the total quantity in the batch. The proportion of contaminated fractions relative to the total mass is higher in the bran or germ compared to the grits. Additionally, variations in mycotoxin contamination levels across different maize batches and unevenness in sampling can influence the final distribution of mycotoxins in various dry-milling fractions.

### 4.2. Wet-Milling

Wet-milling of maize involves steeping the maize in light steep water for 24–48 h, followed by crushing, germ separation, fine grinding, fiber separation, and gluten separation to produce maize starch and various by-products [98]. This fractionation process results in by-products with varying mycotoxin concentrations [99].

#### 4.2.1. Aflatoxins

During the wet-milling process, AFB_1_ is initially transferred to the steeped water. When maize kernels were steeped in water at 25 °C for 6–14 h, the concentration of AFB_1_ was reduced by 15% [100]. Park et al. found that light steep water (LSW) and corn steep liquor (CSL) contained relatively high levels of AFB_1_, at 0.7 and 0.3 µg/kg, respectively [99]. Subsequent studies have modified the steeping conditions of maize kernels. For instance, a 65% reduction in AFB_1_ was observed after steeping maize kernels in 1% ammonium persulphate for 14 h. Different solutions may alter the structure of AFs [100]. It was also found that the temperature and pH of the steeping solution affect the fate of mycotoxins. Generally, the concentration of AFs decreases as the temperature of the steeping solution increases. Additionally, AFs decrease more rapidly in solutions with a pH of 4 compared to neutral pH [100]. Yahl et al. steeped maize kernels in a dilute lactic acid solution and SO_2_ (pH = 4) at 49 °C for 48 h and observed a significant accumulation of AFB_1_ in the steeped water, with only 1–1.2% of AFB_1_ accumulating in the starch fraction [101]. Okeke et al. reported that AFB_1_ and AFB_2_ were reduced by approximately 40–88% and 70–95% in the starch fraction, respectively [102]. The concentration of AFB_1_ in the protein fraction was 17–20%, and it was also higher in the fiber fraction [101].

#### 4.2.2. Ochratoxin

Wood steeped maize kernels in a solution of 0.15% SO_2_ and observed a 37% reduction in OTA levels and a 95% reduction in OTB levels [103]. Compared to raw maize, the levels of OTA and OTB in the endosperm fraction were reduced by approximately 53% and 81%, respectively. In the unseparated endosperm and fiber fractions, OTA and OTB levels were reduced by around 50% and 94%, respectively. Relatively high levels of these mycotoxins accumulate in the liquid fractions [99].

#### 4.2.3. Fusarium Toxins

FBs accumulated in both liquid fractions and solid by-products during wet-milling. The highest levels were found in maize gluten, while the germ and bran fractions had lower concentrations [99]. For example, there were 2240.8 µg/kg of FBs in maize gluten, 1555.0 µg/kg in germ, and 1097.7 µg/kg in bran [99]. FBs are soluble in water and can be transferred to maize gluten feed via LSW and CSL. The study found that a sodium bisulfite solution delayed the transfer of FBs to the liquid fraction [104]. Moreover, FB_1_ and FB_2_ were markedly reduced in the starch [105] and could only be detected in some samples [99].

The distribution of ZEN in commercial wet-milling components has become increasingly widespread. The ZEN content in the fractions obtained through wet-milling follows the order gluten > solubles > fiber > germ [106]. Gluten contains approximately 2–8 times more ZEN than the original maize [93,107]. Between 17 and 26% of ZEN is found in the solubles of the steep water, wash water, and filtrates [106]. Although ZEN is relatively insoluble in water, its solubility increases with higher pH levels [11,108]. The starch fraction is almost free of detectable ZEN levels [93]. However, trace amounts of ZEN have also been detected in starch fractions in some studies. For instance, Park et al. reported trace amounts of ZEN in starch (7.9 and 7.8 µg/kg), representing an average reduction of 80 to 90% compared to raw maize [99,105].

A significant accumulation of water-soluble DON occurs in light steep liquor (LSL), LSW, and CSL after commercial wet-milling. Some of the DON is converted to DON-3-glucoside (DON-3-Glc), and 3-acetyl-DON accumulates in the steeping water. The contamination in the solid fraction (germ, fiber, and gluten) is 5–10 times lower than in raw maize [11,107]. Additionally, DON is reduced in bran fractions by approximately 85–100% [105]. Starch is less contaminated by DON in the wet-milling fraction, with an average reduction of approximately 99% or more [99].

The highest concentration of T-2 toxin in solubles is approximately 10 times higher than in raw kernels, and the liquid component also contains a certain amount of T-2 toxin [109]. The levels of T-2 toxin in the germ increased by around 76% [110]. The lowest concentration was found in the starch, which was approximately 92% lower compared to the original maize [109]. The concentrations of T-2 toxin in the fiber were around 25% lower, with the mean T-2 levels in the gluten remaining nearly unchanged. Additionally, a reduction of about 83–100% was observed in bran fractions [109].

We summarized the fate of fusarium toxins during the wet-milling process. FBs are significantly reduced in the starch fraction, present at lower levels in the germ fraction, and most concentrated in the steeping solution. ZEN is largely undetectable in the starch fraction. The highest levels of ZEN are found in gluten, where levels can be up to about eight times higher than in raw maize, with relatively high levels also present in the fiber and germ fractions. Additionally, the majority of DON is transferred to the steeping water.

## 5. Strategies to Control Mycotoxins in Maize During the Pre-Harvest Period

### 5.1. Pretreatment

Caution must be exercised during maize transportation, with particular attention to the use of appropriate transportation equipment to prevent crop damage, which can lead to fungal invasion and subsequent mycotoxin contamination. Damaged maize should be promptly removed upon detection [111]. After harvest, maize should be dried to a safe moisture content of 10–13% to inhibit fungal growth and mycotoxin formation [112]. Cleaning maize before storage can significantly reduce mycotoxin contamination by sorting and removing visibly moldy, damaged, and inadequately covered husks [113]. By segregating discolored grains that may be contaminated with fungi, the risk of mycotoxin production is minimized [53].

### 5.2. Storage

The proposed strategies to control mycotoxins during the storage of maize are crucial for the maize industry. Proper storage facilities are essential for minimizing the production of mycotoxins after harvest. By maintaining dry and clean conditions, and controlling temperature, humidity, ventilation, and other environmental factors in storage equipment, the growth of molds and the production of mycotoxins can be effectively inhibited. Ensuring appropriate storage conditions [114], whether in well-ventilated facilities or sealed storage bags, is essential for minimizing mycotoxin contamination. It is crucial to maintain hygiene and properly manage storage containers [115], and to employ decontamination methods when necessary [116]. Furthermore, effective insect control will also significantly reduce the levels of different mycotoxins [116,117].

### 5.3. Processing

The process of maize processing to produce various components carries the risk of mycotoxin contamination. Therefore, it is crucial to implement strategies to control mycotoxins during maize processing. Mycotoxins can be managed through physical separation, chemical inactivation, and biodegradation methods that occur during the processing stage [118]. Determining pre-process mycotoxin levels is a crucial aspect of mycotoxin management. Establishing appropriate sampling procedures during this process is essential to ensure that the results are reliable and accurate [5].

Minimizing sampling errors and confounding factors related to sample representativeness can lead to more reliable mycotoxin analyses and accurate mycotoxin distributions. Therefore, accurate sampling is crucial [4]. Accurate determination of mycotoxin species and content is also necessary for effective monitoring of mycotoxins and the implementation of appropriate detoxification measures during processing [118]. The development of specialized mycotoxin sorters has garnered attention among scientists involved in the cleaning and decontamination process. Improved gravity and optical sorters have proven effective in reducing levels of FBs and AFs in maize [119].

#### 5.3.1. Dry-Milling

Firstly, the genotype of the maize must be controlled, as studies have shown that the presence of transgenic traits can affect the fate of FBs during industrial dry-milling [120]. Different degerming processes during dry-milling can lead to varying health risks associated with FB contamination in the endosperm fractions, so it is crucial to select an appropriate dry-milling degerming process [121]. Furthermore, research suggests that the mycotoxin content is inversely related to the size of the dry-milled particles [122], indicating that the final size of the dry-milled particles should be carefully controlled for different types of mycotoxins. To investigate the dry-milling process, multivariate techniques, such as mathematical modeling in experimental design, can be utilized to optimize the reduction of mycotoxins in various fractions [85]. Additionally, characterizing and manipulating kernel properties and dry-milling practices can further mitigate mycotoxin contamination in dry-milled fractions [4].

#### 5.3.2. Wet-Milling

The wet-milling process necessitates the incorporation of SO_2_ and lactic acid during steeping. SO_2_ facilitates starch–protein separation and inhibits microbial proliferation, thereby reducing AF content in maize. Consequently, the concentration of SO_2_ must be meticulously controlled [103]. Previous studies have demonstrated that optimal conditions for maximizing AF reduction involve a steeping duration of 18 h and a lactic acid concentration of 1.0% (*w/v*) [85]. Therefore, the addition of lactic acid must be carefully managed during steeping, alongside the regulation of steeping temperature and duration. Furthermore, lactic acid bacteria with distinct detoxification capabilities can be selected based on the type of mycotoxin present and utilized in combination to enhance degradation efficiency [118]. The milling process can contribute to reducing mycotoxin levels in the endosperm, as the outer layer of the grain is more prone to mycotoxin contamination [85]. Consequently, the grinding granularity, duration, and throughput must be appropriately controlled. Additionally, techniques that facilitate the separation of proteins from starch can be employed to minimize mycotoxin content in starch products. For instance, an ultrasound-assisted maize wet-milling experiment utilized ultrasound to enhance starch–protein separation [123].

The reduction efficiency of mycotoxin concentrations during processing is influenced by various factors, including batch-related variables, mycotoxin characteristics, and processing procedures. Consequently, the implementation of best practices and the monitoring of mycotoxin levels are crucial. Furthermore, process optimization and technological advancements can further mitigate mycotoxin exposure [11]. Maize-processed products must undergo testing before market entry and adhere to maximum residue levels to ensure safety [118]. Additionally, the distribution of mycotoxins across maize fractions is governed by multiple regulatory factors, potentially exhibiting mycotoxin-specific patterns with intricate mechanisms.

## 6. Conclusions

This article provides a comprehensive overview of the occurrence and fate of mycotoxins in maize during the post-harvest period. Future research on the prevalence of mycotoxins in agricultural settings presents significant challenges, as the occurrence of mycotoxins is typically influenced by a complex interplay of various factors. An extensive investigation into the mechanisms underlying the relationship between pathogenic bacteria and mycotoxins should be prioritized. Rapid and accurate early warning systems for mycotoxins should be developed using technologies such as the Internet of Things and Big Data. This approach aims to achieve real-time monitoring and early warning. The variations in toxicity, stability, and production conditions of mycotoxins complicate the harvesting and storage processes. Existing prevention and control technologies may be limited by factors such as cost, efficiency, and other practical considerations, which can impede their large-scale implementation. Future research should emphasize the development of scientific management strategies and intelligent systems for harvesting and storage to enable real-time monitoring of environmental conditions and facilitate timely interventions. Mycotoxins are easily transferred to processing components during the milling process, thereby posing a substantial risk to consumer health. Consequently, addressing mycotoxin contamination and maintaining the quality stability of maize products constitutes a substantial challenge for future research endeavors. Processing conditions and process water must be strictly controlled and managed, while processing techniques should be optimized and new technologies explored. The reduction of mycotoxin contamination in maize is anticipated to significantly enhance food safety and safeguard human health.

## Data Availability

No new data were created.

## Supplementary Materials

The following supporting information can be downloaded at: https://www.mdpi.com/article/10.3390/toxins16110459/s1, Table S1: Types of common mycotoxins in maize; Table S2: Fate of mycotoxins in maize dry-milling process; Table S3: Fate of mycotoxins in maize wet-milling process. Refs. [4,82,84,85,90,94,99,102,105,106,107,109,110,121,124,125,126,127,128,129,130,131,132,133,134,135,136] are cited in Supplementary Materials.
