# Supplementary material for: Occurrence and Fate Analysis of Mycotoxins in Maize During the Post-Harvest Period
Source: Toxins (Basel). 2024 Oct 26;16(11):459. doi: 10.3390/toxins16110459 (PMC11598020; doi:10.3390/toxins16110459)
Supplement: Supplementary file 1 [file toxins-16-00459-s001.zip › toxins-3223896-supplementary.pdf]

Supplementary Materials: Occurrence and Fate Analysis of Mycotoxins in Maize During the Post-Harvest Period

Yajie Zheng , Wenfu Wu, Changpo Sun, Hujun Liu and Jianpeng Dou

Table S1. Types of common mycotoxins in maize.

Mycotoxins	Sources	Structural formula	Toxic effects and disease	Reference
Aflatoxins	*Aspergillus flavus*, *Aspergillus parasiticus*	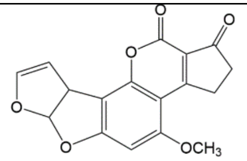	Teratogenic, genotoxic, immunosuppressive, and mutagenic.	[1] [2]
Ochratoxins	*Fusarium graminearum*, *Fusarlum roseum*	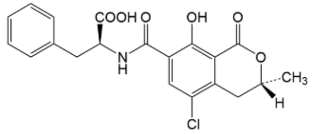	Kidney, renal toxicity, immune organ damage, teratogenicity, and carcinogenicity.	[2] [3] [4]
Deoxynivalenol	*Aspergillus*, *Penicillium*	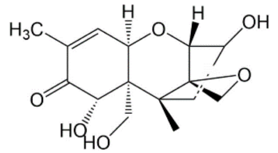	Damage the immune system, embryotoxicity, and teratogenicity.	[5] [6]
Zearalenone	*Fusarium graminearum*, *Fusarium culmorum*, *Fusarium equiseti*	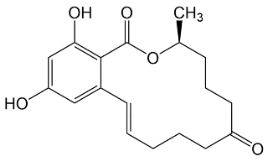	Hyperestrogenism and cervical cancer abnormal reproductive function. Damage to the nervous system, heart, kidneys, liver, and lungs.	[2] [7]
Fumonisins	*Fusarium Moniliform*	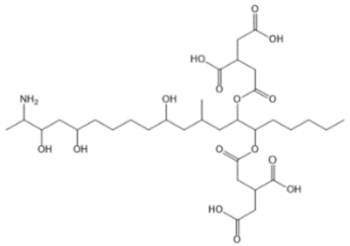	Periventricular leukomalacia, pulmonary edema, hydrothorax, and diseases of the reproductive system.	[8] [9]

Table S2. Fate of mycotoxins in maize dry-milling process.

Mycotoxin	Milling procedure	Type of maize	Initial level in whole kernels	Fate	Scale	Analysis	Reference
AFB_1	Industrial dry-milling coupled with cleaning	FAO 600 class hybrids	Maize 1: 3.6 μg/kg; maize 2: 91.1 μg/kg	Maize 1: 7.5 μg/kg in bran, 9.8 μg/kg in germ, 2.7 μg/kg in coarse grits, 2.3 μg/kg in fine grits, and 3.4 μg/kg in animal feed flour. Maize 2: 189.4 μg/kg in bran, 287.9 μg/kg in germ, 7.6 μg/kg in coarse grits, 7.8 μg/kg in fine grits, and 1.1 μg/kg in animal feed flour.	Factory	HPLC-FLD, HPLC-MS/MS	[10]
	Milling with cleaning (moisture adjustment to 23.5%)	From a local farm in Matelandia in 2016	Not further specified	Yet 38% was present in the endosperm, 54.5% in the pericarp, and 6.7% in the germ.	Laboratory	HPLC-FLD, HPLC-MS	[11]
	Laboratory mill (a hand mill for dehulling)	MM 604, clean white shelled dent corn, from Chalimbana	911.2 μg/kg	1219.4 μg/kg in bran and 151.2 μg/kg in endosperm.	Laboratory	TLC	[12]
AFB_2	Milling with cleaning (moisture adjustment to 23.5%)	From a local farm in Matelandia in 2016	Not further specified	Yet 74% was present in the endosperm, 16% in the pericarp, and 9.9% in the germ.	Laboratory	HPLC-FLD, HPLC-MS	[11]
	Industrial dry-milling coupled	FAO 600 class hybrids	3.4 μg/kg	7.3 μg/kg in bran, 16.7 μg/kg in germ, 0.4 μg/kg in coarse grits, 0.7 μg/kg in fine grits,	Factory	HPLC-FLD,	[10]

	with cleaning			and 8.8 μg/kg in animal feed flour.		HPLC-MS/MS	
	laboratory mill (a hand mill for dehulling)	MM 604, clean white shelled dent corn, from Chalimbana	95.6 μg/kg	119.4 μg/kg in bran and 23.6 μg/kg in endosperm.	Laboratory	TLC	[12]
DON	An experimental dry-milling plant utilizing a modified Buhler MLU-202 laboratory mill (include cleaning)	Uncleaned white maize consignments, from various maize growing localities within South Africa during the 2010 harvest season	Mean: 329.4 μg/kg (range: 68–787 μg/kg)	Mean: 941.4 μg/kg (240-2295 μg/kg) in total hominy feed.	Laboratory	LC-MS/MS	[13]
	Industrial dry-milling	Two different lots (not further specified)	Batch 1：270 μg/kg; Batch 2：472 μg/kg	Batch 1: 1,877 μg/kg in bran, 76 μg/kg in grits, and 886 μg/kg in germ. Batch 2: 6,682 μg/kg in bran, 145 μg/kg in grits, and 2,543 μg/kg in germ.	Factory	HPLC-FLD, HPLC-UV/DAD	[14]
ZEN	An experimental dry-milling plant utilizing a modified Buhler MLU-202 laboratory mill (including cleaning)	Uncleaned white maize consignments, from various maize growing localities within South	Mean: 93.4 μg/kg (range: 8–307 μg/kg)	Mean: 245.6 μg/kg (30-652 μg/kg) in total hominy feed.	Laboratory	LC-MS/MS	[13]

		Africa during the 2010 harvest season					
	Industrial dry-milling	Two different lots (not further specified)	Batch 1：181 μg/kg; Batch 2：123 μg/kg	Batch 1: 855 μg/kg in bran, 42 μg/kg in grits, and 356 μg/kg in germ. Batch 2: 531 μg/kg in bran, 36 μg/kg in grits, and 744 μg/kg in germ.	Factory	HPLC-FLD, HPLC-UV/DAD	[14]
FBs	A commercial dry mill	Not further specified	＜0.1-3.5 μg/g	0.1 μg/g in flaking grits, 0.2-1.1 μg/g in flour, 0.1-2.0 μg/g in germ, and 1.5-3.2 μg/g in bran.	Factory	HPLC-FLD	[15]
FB_1	Industrial dry-milling coupled with cleaning	FAO 600 class hybrids	Maize 1: 5,379 μg/kg; maize 2: 8,841 μg/kg	Maize 1: 7,154 μg/kg in bran, 3,332 μg/kg in germ, 458 μg/kg in coarse grits, 556 μg/kg in fine grits, and 16.011 μg/kg in animal feed flour. Maize 2: 15,647 μg/kg in bran, 11,674 μg/kg in germ, 563 μg/kg in coarse grits, 1,592 μg/kg in fine grits, and 28.712 μg/kg in animal feed flour.	Factory	HPLC-FLD, HPLC-MS/MS	[10]
	A dry-milling technology coupled with a dry degermination (DD) system	From Northern Italy in 2002 to 2006	Mean: 4,580 μg/kg (273-8,480 μg/kg)	11,400 μg/kg (1,310-26,800 μg/kg) in bran, 3,450 μg/kg (175-9,920μg/kg) in germ, 454 μg/kg (305-537 μg/kg) in brewery grits, and 499 μg/kg (85-1,340 μg/kg) in meal.	Factory	HPLC-FLD	[16]
	A dry-milling technology coupled to a DD system and	Cultivated in 2011–2013 period in the same growing area	534 μg/kg	DD system: 159 μg/kg in break meal, 396 μg/kg in germ, 114 μg/kg in pearl meal, 454 μg/kg in corn flour, and 1,718 μg/kg in animal	Factory	LC-MS/MS	[17]

	a tempering-degermination (TD) system	in Northwest Italy		feed flour. DT system: 205 μg/kg in small hominy grits, 386 μg/kg in germ, 109 μg/kg in medium hominy grits, 54 μg/kg in flaking grits, and 2,069 μg/kg in animal feed flour.			
FB_2	Industrial dry-milling coupled with cleaning	Freshly harvested corn samples from the 2014 (lot 1) and the 2015 (lot 2) in the North of Paraná State	Lot 1: mean: 270.32 μg/kg (188.87–380.48 μg/kg); lot 2: mean: 439.76 μg/kg (235.85–703.56 μg/kg)	Lot 1: 336.05 μg/kg in pericarp, and 524.16 μg/kg in germ. lot 2: 929.26 μg/kg in pericarp, and 1,182.50 μg/kg in germ.	Factory	HPLC-FLD	[18]
	A dry-milling technology coupled to a DD system and a TD system	Cultivated in 2011–2013 period in the same growing area in Northwest Italy	191 μg/kg	DD system: 57 μg/kg in break meal, 132 μg/kg in germ, 43 μg/kg in pearl meal, 169 μg/kg in corn flour, and 872 μg/kg in animal feed flour. DT system: 66 μg/kg in small hominy grits, 124 μg/kg in germ, 38 μg/kg in medium hominy grits, 54 μg/kg in flaking grits, and 2,069 μg/kg in animal feed flour.	Factory	LC-MS/MS	[17]

Table S3. Fate of mycotoxins in maize wet-milling process.

Mycotoxin	Milling procedure	Type of maize	Initial level in whole kernels	Fate	Scale	Analysis	Reference
AFs	Ambient temperature (29-33 °C) for fermentation on the steeping durations (48, 72, and 96 h)	Yellow maize varieties that had been stored for one and six months, respectively	588 μg/kg	For 48 h: 2.4 μg/kg in steep/wash liquor, and 64 μg/kg in *Ogi* (starch), for 72 h: 4.3 μg/kg in steep/wash liquor, and 87 μg/kg in *Ogi*, for 96 h: 4.5 μg/kg in steep/wash liquor, and 78 μg/kg in *Ogi*.	Laboratory	LC-MS/MS	[19]
	Laboratory wet-milling (including steeping of kernels in water for 48 h)	Not further specified	487 μg/kg	42.3 μg/kg in starch, 123.9 μg/kg in gluten, 56.4 μg/kg in germ and fiber, and 29.2 μg/kg in water process.	Laboratory	HPLC-FLD	[20]
AFB_1	Laboratory wet-milling (including steeping of kernels in water for 48 h)	From local markets in Cairo, Egypt	Not further specified， 120.66 μg/kg in steeped corn	19.30 μg/kg in starch, 45.68 μg/kg in gluten, 15.56 μg/kg in germ and fiber, and 40.12 μg/kg in water process.	Laboratory	HPLC-FLD	[21]
AFB_2	Ambient temperature (29-33 °C) for fermentation on the steeping durations (48, 72, and 96 h)	White and yellow maize varieties that had been stored for one and six months, respectively	White maize：68 μg/kg， yellow maize：456 μg/kg	White maize for 48 h: 10.4 μg/kg in steep/wash liquor, and 15 μg/kg in *Ogi*, for 72 h: 41 μg/kg in steep/wash liquor, and 47 μg/kg in *Ogi*, for 96 h: 30 μg/kg in steep/wash liquor, and 39 μg/kg in *Ogi*.<br>Yellow maize for 48 h: 13 μg/kg in steep/wash liquor, and 44 μg/kg in *Ogi*, for 72 h: 20 μg/kg in steep/wash liquor, and 86 μg/kg in *Ogi*. for 96 h: 29 μg/kg in steep/wash	Laboratory	LC-MS/MS	[19]

				liquor, and 64 μg/kg in *Ogi*.			
ZEN	Laboratory wet-milling (including steeping of kernels in dilute solutions of lactic acid and SO_2, pH 4, at 49°C for 48 h)	Different batches of yellow maize	900-9,400 μg/kg	The content in the germ is 1-2 times higher than the whole maize, the fiber is 1-3 times higher, the solubles contain 1-4 times, and the highest content is found in gluten, which is 2-7 times higher than in the original maize.	Laboratory	TLC	[22]
	Wet-milling in a New Zealand facility	The source of maize being processed during each period was New Zealand, New Zealand, and the USA, respectively (not further specified)	Period 21-25 November: 500 μg/kg, period 1-7 December: 250 μg/kg	Neither the LSL nor CSL contained detectable levels of ZEN, in solid fractions (germ, fiber, and gluten) were two or three times higher than in the input maize, and maize oil was found to contain a high concentration (4-6 mg/kg) of ZEN.	Factory	HPLC-UV, HPLC-FLD	[23]
DON	Ferment spontaneously at ambient temperature (range: 27–30 °C) for 72 h. then the water was drained and the maize grains were milled	From a market in Kaduna town (Kaduna State, Nigeria)	99 μg/kg	61 μg/kg in 36 h fermented maize, 33 μg/kg in 72 h fermented maize, not detected in *Ogi*.	Laboratory	UPLC-MS	[24]

	Commercial wet-milling in three Korean mills	Not further specified	Mean: 213.6 µg/kg (LOD–833.7 µg/kg)	With an average reduction of approximately 99% (or more) in starch. The average levels in LSW and CSL were 3,641.2 and 7,417.5 µg/kg, respectively.	Factory	LC-MS	[25]
FBs	Ambient temperature (29-33 °C) for fermentation on the steeping durations (48, 72 and 96 h)	White and yellow maize varieties that had been stored for one and six months, respectively	White maize：780 µg/kg，yellow maize：2,294 µg/kg	White maize for 48 h: 87 µg/kg in steep/wash liquor, and 39 µg/kg in *Ogi*, for 72 h: 376 µg/kg in steep/wash liquor, and 86 µg/kg in *Ogi*, for 96 h: 211 µg/kg in steep/wash liquor, and 66 µg/kg in *Ogi.* Yellow maize for 48 h: 104 µg/kg in steep/wash liquor, and 111 µg/kg in *Ogi*, for 72 h: 181 µg/kg in steep/wash liquor, and 161 µg/kg in *Ogi*. for 96 h: 189 µg/kg in steep/wash liquor, and 128 µg/kg in *Ogi.*	Laboratory	LC-MS/MS	[19]
FB_1	Seeping of kernels in a 0.55% lactic acid, 0.2% sulfurous acid solution at 52°C for 36 h	1989 yellow com and 1990 com screenings, No.2 yellow dent	13,900 µg/kg	About 61% lower in gluten, 70% lower in fiber, and 84% lower in germ.	Laboratory	GC-MS	[26]
FB_2	Commercial wet-milling in three Korean mills	Not further specified	Mean: 15.8 µg/kg (LOD–53.9 µg/kg)	3.0 µg/kg in starch, 824.9 µg/kg in gluten, 512.1 µg/kg in germ, 344 µg/kg in gluten feed, 277.4 µg/kg in bran, and 299.9µg/kg corn steep liquor.	Factory	LC-MS	[25]
T-2	Steeping of kernels in 1.5% lactic acid and 0.25% SO_2, pH 4, at	Different batches of yellow dent maize	502-8,700 µg/kg	About 10 times higher than in the raw kernels in solubles, and in the germ increased by around 76%. 92% lower in the starch, and	Laboratory	GC-FID	[27]

50–52°C for 48 h	around 25% lower in the fiber compared to the original maize.

References

1. Bedard, L.L.; Massey, T.E. Aflatoxin B_1-induced DNA damage and its repair. *Cancer Lett.* **2006**, *241*(2), 174-183. https://doi.org/10.1016/j.canlet.2005.11.018.
2. De Ruyck, K.; De Boevre, M.; Huybrechts, L.; De Saeger, S. Dietary mycotoxins, co-exposure, and carcinogenesis in humans: Short review. *Mutation Research/Reviews in Mutation Research* **2015**, *766*, 32-41. https://doi.org/10.1016/j.mrrev.2015.07.003.
3. Zepnik, H.; Pähler, A.; Schauer, U.; Dekant, W. Ochratoxin A-induced tumor formation: Is there a role of reactive Ochratoxin A metabolites? *Toxicol. Sci.* **2001**, *59*(1), 59-67. https://doi.org/10.1093/toxsci/59.1.59.
4. Bayman, P.; Baker, J.L. Ochratoxins: A global perspective. *Mycopathologia* **2006**, *162*(3), 215-223. https://doi.org/10.1007/s11046-006-0055-4.
5. Hope, R.; Aldred, D.; Magan, N. Comparison of environmental profiles for growth and deoxynivalenol production by *Fusarium culmorum* and *F. graminearum* on wheat grain. *Lett. Appl. Microbiol.* **2005**, *40*(4), 295-300. https://doi.org/10.1111/j.1472-765X.2005.01674.x.
6. Zhang, X.; Jiang, L.; Geng, C.; Cao, J.; Zhong, L. The role of oxidative stress in deoxynivalenol-induced DNA damage in $HepG_2$ cells. *Toxicon* **2009**, *54*(4), 513-518. https://doi.org/10.1016/j.toxicon.2009.05.021.
7. Eriksen, G.S.; Alexander, J., Fusarium toxins in cereals - a risk assessment TemaNord **1998**: Copenhagen.
8. Mike Bolger, R.D.C., Michael DiNovi, David Gaylor, Wentzel Gelderblom, Monica Olsen, Nachman Paster, Ronald T. Riley, Gordon Shephard and Gerrit J.A. Speijers. Fumonisins: Safety evaluations of certain mycotoxins in foods. *FAO Food Nutr.* **2001**, *74*, 103-279. http://www.inchem.org/documents/jecfa/jecmono/v47je03.htm.
9. Weidenbörner, M. Foods and fumonisins. *Eur. Food Res. Technol.* **2001**, *212*(3), 262-273. https://doi.org/10.1007/s002170000259.
10. Pietri, A.; Zanetti, M.; Bertuzzi, T. Distribution of aflatoxins and fumonisins in dry-milled maize fractions. *Food Addit. Contam., Part A: Chem., Anal., Control, Exposure Risk Assess.* **2009**, *26*(3), 372-380. https://doi.org/10.1080/02652030802441513.
11. Massarolo, K.C.; Rodrigues, P.; Ferreira, C.F.J.; Kupski, L.; Badiale-Furlong, E. Simultaneous distribution of aflatoxins B_1 and B_2, and fumonisin B_1 in corn fractions during dry and wet-milling. *J. Food Sci. Technol.* **2022**, *59*(8), 3192-3200. https://doi.org/10.1007/s13197-022-05373-9.
12. Njapau, H.; Muzungaile, E.M.; Changa, R.C. The effect of village processing techniques on the content of aflatoxins in corn and peanuts in Zambia. *J. Sci. Food Agric.* **1998**, *76*(3), 450-456. https://doi.org/10.1002/(SICI)1097-0010(199803)76:3<450::AID-JSFA970>3.0.CO;2-L.
13. Burger, H.M.; Shephard, G.; Louw, W.; Rheeder, J.; Gelderblom, W. The mycotoxin distribution in maize milling fractions under experimental conditions. *Int. J. Food Microbiol.* **2013**, *165*, 57-64. https://doi.org/10.1016/j.ijfoodmicro.2013.03.028.
14. Schollenberger, M.; Müller, H.-M.; Rüfle, M.; Suchy, S.; Drochner, W. Redistribution of 16 Fusarium toxins during commercial dry milling of maize. *Cereal Chem.* **2008**, *85*(4), 557-560. https://doi.org/10.1094/CCHEM-85-4-0557.
15. Katta, S.K.; Cagampang, A.E.; Jackson, L.S.; Bullerman, L.B. Distribution of Fusarium molds and Fumonisins in dry-milled corn fractions. *Cereal Chem.* **1997**, *74*(6), 858-863. https://doi.org/10.1094/CCHEM.1997.74.6.858.
16. Vanara, F.; Reyneri, A.; Blandino, M. Fate of fumonisin B_1 in the processing of whole maize kernels during dry-milling. *Food Control* **2009**, *20*(3), 235-238. https://doi.org/10.1016/j.foodcont.2008.05.014.
17. Vanara, F.; Scarpino, V.; Blandino, M. Fumonisin distribution in maize dry-milling products and by-products: Impact of two industrial degermination systems. *Toxins* **2018**, *10*(9), 357. https://doi.org/10.3390/toxins10090357
18. Bordini, J.G.; Ono, M.A.; Garcia, G.T.; Fazani, V.H.M.; Vizoni, É.; Rodrigues, K.C.B.; Hirooka, E.Y.; Ono, E.Y.S. Impact of industrial dry-milling on fumonisin redistribution in non-transgenic corn in Brazil. *Food Chem.* **2017**, *220*, 438-443. https://doi.org/10.1016/j.foodchem.2016.10.028.
19. Okeke, C.A.; Ezekiel, C.N.; Sulyok, M.; Ogunremi, O.R.; Ezeamagu, C.O.; Šarkanj, B.; Warth, B.; Krska, R. Traditional processing impacts mycotoxin levels and nutritional value of ogi - A maize-based complementary food. *Food Control* **2018**, *86*, 224-233. https://doi.org/10.1016/j.foodcont.2017.11.021.
20. Aly, S.E. Distribution of aflatoxins in product and by-products during glucose production from contaminated corn. *Food/Nahrung* **2002**, *46*(5), 341-344. https://doi.org/10.1002/1521-3803(20020901)46:5<341::AID-FOOD341>3.0.CO;2-N.

21. Aly, S.E.; Hathout, A.S. Fate of aflatoxin B_1 in contaminated corn gluten during acid hydrolysis. *J. Sci. Food Agric* **2011**, *91*(3), 421-427. https://doi.org/10.1002/jsfa.4201.
22. Bennett, G.A.; Vandegraft, E.E.; Shotwell, O.L.; Watson, S.A.; Bocan, B.J. Zearalenone: distribution in wet-milling fractions from contaminated corn. *Cereal Chem.* **1978**, *55*, 455-461.
23. Lauren, D.R.; Ringrose, M.A. Determination of the fate of three Fusarium mycotoxins through wet-milling of maize using an improved HPLC analytical technique. *Food Addit. Contam.* **1997**, *14*(5), 435-443. https://doi.org/10.1080/02652039709374549.
24. Chilaka, C.A.; De Boevre, M.; Atanda, O.O.; De Saeger, S. Fate of Fusarium mycotoxins during processing of Nigerian traditional infant foods (ogi and soybean powder). *Food Res. Int.* **2019**, *116*, 408-418. https://doi.org/10.1016/j.foodres.2018.08.055.
25. Park, J.; Kim, D.H.; Moon, J.Y.; An, J.A.; Kim, Y.W.; Chung, S.H.; Lee, C. Distribution analysis of twelve mycotoxins incorn and corn-derived products by LC-MS/MS to evaluate the carry-over ratio during wet-milling. *Toxins* **2018**, *10*(8), 319. https://doi.org/10.3390/toxins10080319.
26. Bennett, G.A.; Richard, J.L.; Eckhoff, S.R. Distribution of fumonisins in food and feed products prepared from contaminated corn. *Adv. Exp. Med. Biol* **1996**, *392*, 317-322. https://doi.org/10.1007/978-1-4899-1379-1_27.
27. Collins, G.J.; Rosen, J.D. Distribution of T-2 Toxin in wet-milled corn products. *J. Food Sci.* **1981**, *46*(3), 877-879. https://doi.org/10.1111/j.1365-2621.1981.tb15370.x.
